# Supplementary material for: Complexome profiling of the Chlamydomonas psb28 mutant reveals TEF5 as an early PSII assembly factor
Source: Plant Cell. 2025 Mar 17;37(6):koaf055. doi: 10.1093/plcell/koaf055 (PMC12164590; doi:10.1093/plcell/koaf055)
Supplement: koaf055_Supplementary_Data [file koaf055_supplementary_data.zip › psb28_interactive.zip!/plots/intensity/qualityPlots/peptides/_Cre17.g698450.t1.2.html]

### Cre17.g698450.t1.2

Trivial name:   
SeqCoverage [median]: 9.50  
Mass: 38.10 kDa  
  
 

| Sequence | MissedCleavage | Mass [Da] | wtIntensity | muIntensity | Score |
| --- | --- | --- | --- | --- | --- |
| DAAVTVVHSATPK | 0 | 1294.69 | 620.38 | 515.78 | 88.68 |
| DSQTYVR | 0 | 867.41 | 0.00 | 303.09 | 81.78 |
| SNIVGMPAALLLNKR | 1 | 1595.92 | 1214.46 | 0.00 | 59.19 |
|  |  |  | 1834.8 | 818.9 |  |
